# Enhanced Bioavailability of AC1497, a Novel Anticancer Drug Candidate, via a Self-Nanoemulsifying Drug Delivery System

**DOI:** 10.3390/pharmaceutics13081142

**Published:** 2021-07-27

**Authors:** Kshitis Chandra Baral, Jae-Geun Song, Sang Hoon Lee, Rajiv Bajracharya, Godesi Sreenivasulu, Minkyoung Kim, Kyeong Lee, Hyo-Kyung Han

**Affiliations:** BK21 FOUR Team and Integrated Research Institute for Drug Development, College of Pharmacy, Dongguk University-Seoul, Dongguk-ro-32, Ilsan-Donggu, Goyang 10326, Korea; kshitis087@gmail.com (K.C.B.); dkfmrhtm@gmail.com (J.-G.S.); sh_lee@dongguk.edu (S.H.L.); rajivbajra@hotmail.com (R.B.); sreenivasulu.godesi@gmail.com (G.S.); kyoung2k@naver.com (M.K.); kaylee@dongguk.edu (K.L.)

**Keywords:** AC1497, self-nanoemulsifying drug delivery system, bioavailability, dissolution, solubilization

## Abstract

AC1497 is an effective dual inhibitor of malate dehydrogenase 1 and 2 targeting cancer metabolism. However, its poor aqueous solubility results in low bioavailability, limiting its clinical development. This study was conducted to develop an effective self-nanoemulsifying drug delivery system (SNEDDS) of AC1497 to improve its oral absorption. Based on the solubility of AC1497 in various oils, surfactants, and cosurfactants, Capryol 90, Kolliphor RH40, and Transcutol HP were selected as the components of SNEDDS. After testing various weight ratios of Capryol 90 (20–30%), Kolliphor RH40 (35–70%), and Transcutol HP (10–35%), SNEDDS-F4 containing 20% Capryol 90, 45% Kolliphor RH40, and 35% Transcutol HP was identified as an optimal SNEDDS with a narrow size distribution (17.8 ± 0.36 nm) and high encapsulation efficiency (93.6 ± 2.28%). Drug release from SNEDDS-F4 was rapid, with approximately 80% of AC1497 release in 10 min while the dissolution of the drug powder was minimal (<2%). Furthermore, SNEDDS-F4 significantly improved the oral absorption of AC1497 in rats. The maximum plasma concentration and area under the plasma concentration–time curve of AC1497 were, respectively 6.82- and 3.14-fold higher for SNEDDS-F4 than for the drug powder. In conclusion, SNEDDS-F4 with Capryol 90, Kolliphor RH40, and Transcutol HP (20:45:35, *w*/*w*) effectively improves the solubility and oral absorption of AC1497.

## 1. Introduction

Cancer cells undergo abnormal metabolic processes, including aerobic glycolysis (Warburg effect) and a high rate of glutamine metabolism to produce energy [1,2]. Since these metabolic alterations are closely related to excessive cell proliferation and resistance to chemotherapy [2,3], enzymes involved in cancer cell metabolism are important targets for the development of anticancer drugs. Among various enzymes controlling tumor growth, malate dehydrogenase (MDH) 1 and MDH2 play an important role in cancer cell metabolism; it provides a source of NAD+, a vital cofactor for high aerobic glycolysis [2,3]. MDH1 and MDH2 catalyze the interconversion between malate and oxaloacetate in the tricarboxylic acid (TCA) cycle using the NAD/NADH cofactor system [4]. Therefore, the dual inhibition of MDH1/2 can be a valuable approach for developing novel anticancer drugs suppressing cancer cell metabolism.

**Figure 1:**
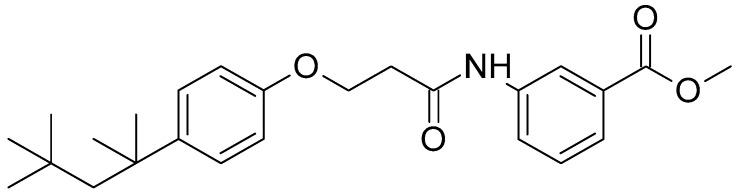
Chemical structure of AC1497.

Recently, AC1497 (methyl 3-(3-(4-(2,4,4-trimethylpentan-2-yl)phenoxy)propanamido)benzoate) (Figure 1) was developed as a dual inhibitor of MDH1/2 targeting cancer metabolism [5]. It was found to effectively inhibit mitochondrial respiration and hypoxia-induced hypoxia-inducible factor (HIF)-1α accumulation, leading to significant antitumor activity [5]. However, owing to its hydrophobic nature, AC1497 has poor aqueous solubility (0.683 µg/mL), which limits its oral bioavailability and in vivo applications. Therefore, to maximize the therapeutic effect of AC1497, effective formulations that improve the solubility and bioavailability of AC1497 are required.

Various formulation approaches have been adopted for enhancing the solubility of drugs, including solid dispersion, nanocrystals, micro/nanoemulsion, and lipid-based drug delivery systems [6]. Among them, the self-nanoemulsifying drug delivery system (SNEDDS) is widely applied to improve the bioavailability of poorly soluble drugs. A SNEDDS comprises isotropic mixtures of oil and surfactant, usually in combination with cosolvents or cosurfactants [7]. Upon exposure to an aqueous medium with gentle shaking, the system undergoes self-emulsification to form an ultrafine oil-in-water (*o*/*w*) nanoemulsion [8,9]. The self-emulsification process occurs spontaneously because the required free energy is very low [10,11]. Therefore, as an orally administered SNEDDS formulation passes through the gastrointestinal (GI) lumen, it comes in contact with aqueous GI fluids and produces nano-sized emulsion droplets under mild agitation provided by GI motility [12]. This spontaneous formation of nanoemulsions in the GI tract retains the drug in the solution state inside the oil droplets. In addition, the nano-sized droplets provide a large interfacial surface area for rapid drug release [13]. SNEDDS can also promote lymphatic drug transport, thus avoiding the hepatic first-pass metabolism. Consequently, SNEDDS has been actively adopted to improve the oral bioavailability of poorly soluble drugs. For example, Nepal et al. [14] improved the oral bioavailability of poorly soluble coenzyme Q_10_ by formulating it into a Witepsol H35-based SNEDDS. Balakumar et al. [15] also developed a SNEDDS of rosuvastatin calcium by using cinnamon oil 30%, Labrasol 60%, and Capmul MCM C8 10%, which enhanced the bioavailability of rosuvastatin by 2.45 folds compared to that of the drug suspension.

SNEDDS enhance the bioavailability of lipophilic drugs by multiple mechanisms, including (i) improving the solubility and membrane permeability of drugs, (ii) enhancing GI drug stability, and (iii) facilitating lymphatic drug absorption [16,17,18]. Therefore, the aim of the present study was to develop an optimal SNEDDS of AC1497 to improve its oral bioavailability.

## 2. Materials and Methods

### 2.1. Materials

AC1497 was obtained from Professor K. Lee (College of Pharmacy, Dongguk University) [5]. Labrafil M 2125CS, Labrafil M 1944 CS, Capryol 90, Labrasol, and Transcutol HP were provided by Gattefossé (Saint-Priest, Lyon, France). Captex 355, Capmul PG8, and Capmul MCM C8 were procured as a gift from ABITEC Corporation (Janesville, WI, USA). Kolliphor ELP, Kolliphor RH 40, and Solutol HS 15 were provided by BASF (Ludwigshafen, Germany). Dulbecco’s Modified Eagle’s Medium (DMEM), nonessential amino acids, fetal bovine serum (FBS), antibiotics (penicillin-streptomycin), and all other reagents used in cell culture studies were obtained from Thermo Fisher Scientific (Waltham, MA, USA). Other chemicals and solvents were purchased from Merck KGaA (Darmstadt, Germany). All solvents were of high-performance liquid chromatography (HPLC) grade. 

Caco-2 cells (human epithelial colorectal adenocarcinoma cells) were purchased from the Korean Cell Line Bank (Seoul, Korea). Caco-2 cells were grown in DMEM containing 10% FBS, 1% nonessential amino acid, and 1% antibiotics. Cells were incubated at 37 °C in an atmosphere of 5% CO_2_ and 90% relative humidity.

### 2.2. Screening of SNEDDS Components

#### 2.2.1. Solubility Studies in Various Oils, Surfactants, and Cosurfactants

The solubility of AC1497 was measured in various oils, surfactants, and cosurfactants by using the shake-flask method [15,19,20]. An excess amount of AC1497 was added to 1 mL of each vehicle (oils: oleic acid, Captex 355, Labrafil M 2125CS, Labrafil M 1944 CS, Capmul MCM C8, Capryol 90, and krill oil; surfactants: Solutol HS15, Tween 80, Brij L4, Labrasol, Kolliphor ELP, and Kolliphor RH40; and cosurfactants: PEG400, propylene glycol, Capmul PG8, ethanol, and Transcutol HP). After vigorous mixing for 2 min, the mixtures were maintained in a shaking water bath at 37 °C for 72 h, followed by centrifugation at 17,000× *g* for 10 min. The supernatants went through membrane filters (pore size: 0.45 µm). Drug concentrations in the filtrates were measured by an HPLC assay as described in Section 2.11.

#### 2.2.2. Emulsification Efficiency of Surfactants and Cosurfactants

The emulsification ability of various surfactants was evaluated in the selected oil (Capryol 90). Each surfactant (250 µL) was added to an equal volume of Capryol 90. The mixtures were heated at 40 °C for 2 min with vigorous mixing. The mixture (20 µL) was diluted to 2 mL with distilled water in a glass-stoppered flask, which was then inverted several times. The frequency of flask inversions necessary for the formation of a clear and uniform nanoemulsion was determined to assess the ease of emulsion formation. In addition, the obtained fine emulsions were kept for 2 h, and the transmittance (%) was measured at 638 nm using a UV-Vis spectrophotometer [21,22]. 

Cosurfactants were also screened to optimize the emulsification ability of surfactants. Briefly, 40 µL of the selected surfactant was mixed with 20 µL of each cosurfactant. Then, the selected oil (60 µL) was added to the mixture and heated at 40 °C for 2 min with vigorous mixing. The mixture (20 µL) was then diluted to 2 mL with distilled water. After dilution, the frequency of flask inversions necessary for the formation of a clear emulsion and the transmittance (%) were determined in the same manner as described above for surfactants.

### 2.3. Construction of a Pseudoternary Phase Diagram

In the initial screening described in Section 2.2., Capryol 90, Kolliphor RH 40, and Transcutol HP were selected as the optimal oil, surfactant, and co-surfactant, respectively, based on their solubilization capacity and emulsification efficiency. These three components were mixed homogeneously at various weight ratios with vortexing for a few minutes. Then, each mixture was titrated with water under gentle agitation at 37 °C. Spontaneous emulsion formation and phase clarity were examined, and the phase diagram was constructed using SigmaPlot^®^ (Systat Software Inc., Palo Alto, CA, USA) to identify the good self-emulsification region [23].

### 2.4. Preparation of Drug-Loaded SNEDDS

Based on the pseudoternary phase diagram, a series of drug-loaded SNEDDS were prepared at various weight ratios of the selected oil (20–30%), surfactant (35–70%), and co-surfactant (10–35%). In all formulations, the drug amount was kept constant. Briefly, the oil, surfactant, and co-surfactant were mixed in stoppered glass vials using a vortex mixer. Then, AC1497 (2% *w*/*w*) was added to the resulting mixture and vortexed for homogenous mixing until a clear solution was obtained. The prepared formulations were stored in sealed transparent vials at room temperature until analyzed.

### 2.5. Cytotoxicity Study

Caco-2 cells were seeded in 96-well plates at a density of 1 × 10^4^ cells/well. After 24 h of incubation, the cells were treated with empty SNEDDS without drugs at different concentrations for 48 h at 37 °C. At the end of incubation, thiazolyl blue tetrazolium bromide was added to each well and incubated for another 4 h. Subsequently, the medium was removed, and 100 μL of DMSO was added to dissolve the formazan crystals. The absorbance of each sample was determined by a microplate reader at 550 nm. The cell viability (%) was calculated and compared with the untreated control.

### 2.6. In Vitro Characterization of SNEDDS

#### 2.6.1. Robustness to Dilution, Self-Emulsification Time, and Percent Transmittance

Each formulation was diluted 10, 100, and 1000 times with distilled water, 0.1 N HCl, or phosphate buffer (pH 6.8) while stirring at 100 rpm at 37 °C to simulate in vivo dilution behavior. Each diluted sample was kept at an ambient temperature for 24 h to monitor for any phase separation or drug precipitation.

For the assessment of self-emulsification time, 1 mL of each formulation was added to 500 mL of distilled water and gently mixed at 37 °C while stirring at 100 rpm. The emulsification time was assessed visually as the time required to form a clear emulsion upon dilution. The transmittance (%) of each SNEDDS was also measured as a determinant of optical clarity by using a UV-Vis spectrophotometer at 638 nm.

#### 2.6.2. Droplet Size, Zeta Potential, and Morphology

Each formulation (0.2 mL) was diluted with 20 mL of distilled water. The average droplet size and zeta potential of the obtained nanoemulsion were measured by dynamic light scattering (DLS) using a Zetasizer Nano-ZS90 (Malvern Instruments, Malvern, UK). The polydispersity index (PDI) was also obtained to assess the size distribution. 

The morphological characteristics of the optimized SNEDDS (SNEDDS-F4) were examined using transmission electron microscopy (TEM) (JEM-2100F; JEOL Ltd., Tokyo, Japan) at Korea Basic Science Institute (Chuncheon Center, Korea).

### 2.7. In Vitro Drug Release Studies

Drug release studies of pure drug powder and SNEDDS-F4 were performed in a dissolution tester DT1420 (ERWEKA, Heusenstamm, Germany) at 37 ± 0.5 °C and 50 rpm. Each formulation (equivalent to 5 mg of AC1497) was filled in hard gelatin capsules, which were then exposed to the dissolution medium at pH 1.2–6.8 or distilled water. Samples (1 mL) were withdrawn at predetermined time points (10, 15, 20, 30, 45, and 60 min) and then passed through a membrane filter (pore size: 0.45 µm). An equal volume of fresh medium was added to the vessel to maintain a constant volume of dissolution medium. The released drug amount was determined by HPLC assay.

### 2.8. Drug Release in Simulated Intestinal Fluids

Drug release profiles of pure drug powder and SNEDDS-F4 were examined in the fasted-state simulated intestinal fluid (FaSSIF) and the fed-state simulated intestinal fluid (FeSSIF). The FaSSIF and FeSSIF were prepared as described previously [24,25,26]. Briefly, FaSSIF was composed of 3 mM sodium taurocholate, 0.2 mM lecithin, 19.12 mM maleic acid, 34.8 mM sodium hydroxide, and 68.62 mM sodium chloride. FeSSIF was composed of 10 mM sodium taurocholate, 2 mM lecithin, 55.02 mM maleic acid, 81.65 mM sodium hydroxide, 125.5 mM sodium chloride, 5 mM glyceryl monooleate, and 0.8 mM sodium oleate. The pH was adjusted to 6.5 and 5.8 for FaSSIF and FeSSIF, respectively. Drug release from the drug powder and SNEDDS-F4 was evaluated in FaSSIF and FeSSIF at 37 ± 0.5 °C and 50 rpm, as described in Section 2.7. The collected samples at predetermined time points (0.5, 1, 2, 4, 6, and 8 h) were passed through a syringe filter (pore size: 0.45 µm) and analyzed by HPLC assay.

### 2.9. Stability Studies

Thermodynamic stability studies were conducted to evaluate the effect of centrifugation and temperature on the stability of SNEDDS. Each formulation was diluted with water (1:100) and centrifuged at 5000 rpm for 0.5 h to determine its stability as an isotropic single-phase system. In heat–cool cycles, each SNEDDS was subjected to six cycles between 4 °C and 40 °C with storage at each temperature for 48 h. SNEDDS also underwent three consecutive freezing–thawing cycles at −20 °C and 25 °C with storage at each temperature for 48 h. The physical appearance and phase separation were examined at the end of each cycle.

### 2.10. Pharmacokinetic Study

The pharmacokinetic profiles of pure drug powder and SNEDDS-F4 were examined in rats. The study protocol was approved by the Institutional Animal Care and Use Committee of Dongguk University (IACUC-2017-016-4). Male Sprague Dawley rats (230–250 g, 8 weeks) were purchased from Orient Bio Co., Ltd. (Seongnam, Korea). Rats were housed at 21–22 °C under a 12 h light/dark cycle for 7 days before the experiments. All rats were provided free access to water and a normal standard chow diet (Superfeed Company, Wonju, Korea). Rats were divided into two groups (*n* = 3 per group) and then fasted for 12 h before drug administration. On the day of experiment, the mixture of Alfaxan^®^ and Rompun^®^ (3:1, *v*/*v*) was administered to rats via intramuscular injection for anesthesia. AC1497 was suspended in 0.5% aqueous methylcellulose with 5% polyethylene glycol (PEG). Each formulation (drug suspension or SNEDDS-F4) was administered orally at a dose equivalent to 20 mg/kg of AC1497. Blood samples (300 µL) were collected from the femoral artery at predetermined time points (0.5, 1, 2, 4, 8, 12, and 24 h). Blood samples were centrifuged at 17,000× *g* for 5 min, and the obtained plasma samples were kept frozen at −20 °C until analyzed by liquid chromatography-tandem mass spectrometry (LC-MS/MS).

### 2.11. Analytical Methods

#### 2.11.1. HPLC Assay

Quantification of AC1497 from in vitro samples was performed using an HPLC system (Perkin Elmer series 200, Waltham, MA, USA) and a reversed-phase C_18_ column (Gemini C18, 4.6 × 150 mm, 5 µm; Phenomenex, CA, USA). The mobile phase consisted of acetonitrile and 0.1% trifluoroacetic acid in water (80:20, *v*/*v*). The flow rate was 1.0 mL/min at 30 °C and UV wavelength set at 254 nm. Simvastatin was used as an internal standard (IS), and the calibration curve of AC1497 was linear over the concentration range of 1–100 µg/mL.

#### 2.11.2. LC-MS/MS

Plasma concentrations of AC1497 were determined by LC-MS/MS. Briefly, 100 µL plasma was mixed with 20 µL simvastatin (IS, 10 µg/mL) and 180 µL methanol. After vortexing, each sample was centrifuged at 17,000× *g* for 10 min, and 240 µL of supernatant was dried under a vacuum. The residue was reconstituted with 80 µL of 50% acetonitrile and analyzed by LC-MS/MS. Chromatographic separation was performed with a C_18_ column (4.6 × 100 mm, 2.6 µm; Phenomenex). Mobile phase consisted of 0.1 % formic acid in acetonitrile and 0.1% formic acid in water (85:15, *v*/*v*) and was eluted at a flow rate of 1.0 mL/min. Mass spectrometric detection was performed using the AB Sciex API 4000 triple quadrupole mass spectrometer (AB Sciex, MA, USA). The electrospray ionization (ESI) source was set in the positive ionization mode for AC1497 and simvastatin (IS). The precursor/product ion pair (*m*/*z*) was 412.1 → 149.1 for AC1497. The calibration curve of AC1497 was prepared at concentrations of 0.25–100 ng/mL with good linearity (r^2^ > 0.99).

### 2.12. Pharmacokinetic and Statistical Analysis

Noncompartmental pharmacokinetic analysis was performed using WinNonlin^®^ (Pharsight Co., Mountain View, CA, USA). The area under the plasma concentration–time curve (AUC) was estimated by the linear trapezoidal method. The maximum plasma concentration (C_max_) and time to reach the maximum plasma concentration (T_max_) were directly observed values from the plasma concentration–time curves. 

All the data are expressed as the mean ± standard deviation (SD). Statistical analysis was performed using Student’s t-test, and a *p* value less than 0.05 was considered statistically significant.

## 3. Results and Discussion

### 3.1. Screening of SNEDDS Components

**Figure 2:**
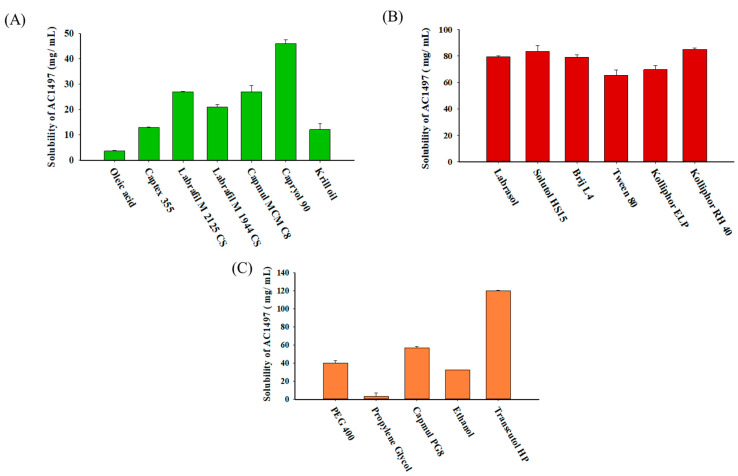
Solubility of AC1497 in various oils (**A**), surfactants (**B**), and cosurfactants (**C**) (mean ± SD, *n* = 3).

To develop an effective SNEDDS, the components (oil, surfactant, and cosurfactant) of the ternary system were selected based on their solubilization capacity and emulsification efficiency. The solubilization capacity of the oil phase is critical to prevent drug precipitation upon dilution, and it also affects drug loading efficiency [16,17]. Among the tested oils, Capryol 90, a medium-chain fatty acid, enhanced the solubility of AC1497 to the greatest extent (Figure 2A). Therefore, Capryol 90 was selected as an optimal oil for SNEDDS formulations. Subsequently, various surfactants were also examined for their solubilization capacity and emulsification efficiency. Considering that (i) non-ionic surfactants exhibit less toxicity and lower critical micelle concentration than ionic surfactants [10,27], and (ii) surfactants having high hydrophilic–lipophilic balance (HLB) values can form a fine and uniform emulsion under mild agitation [14], the emulsification efficiency of non-ionic surfactants having HLB > 10 was examined for the formation of o/w nanoemulsions. As summarized in Table 1 and Figure 2B, Kolliphor RH 40 (HLB: 14–16) [28] effectively improved the drug solubility and showed the highest percentage of transmittance upon dilution. In addition, Transcutol HP as a cosurfactant exhibited the highest solubilization effect and required the minimum number of flask inversions to form a transparent emulsion (Table 1 and Figure 2C). These results suggest that the incorporation of Transcutol HP as a cosurfactant could help improve drug loading and the formation of an extemporaneous nanoemulsion. Therefore, Capryol 90, Kolliphor RH 40, and Transcutol HP were selected as the oil, surfactant, and cosurfactant, respectively, to construct a pseudoternary phase diagram.

### 3.2. Cytotoxicity of SNEDDS Components

**Figure 3:**
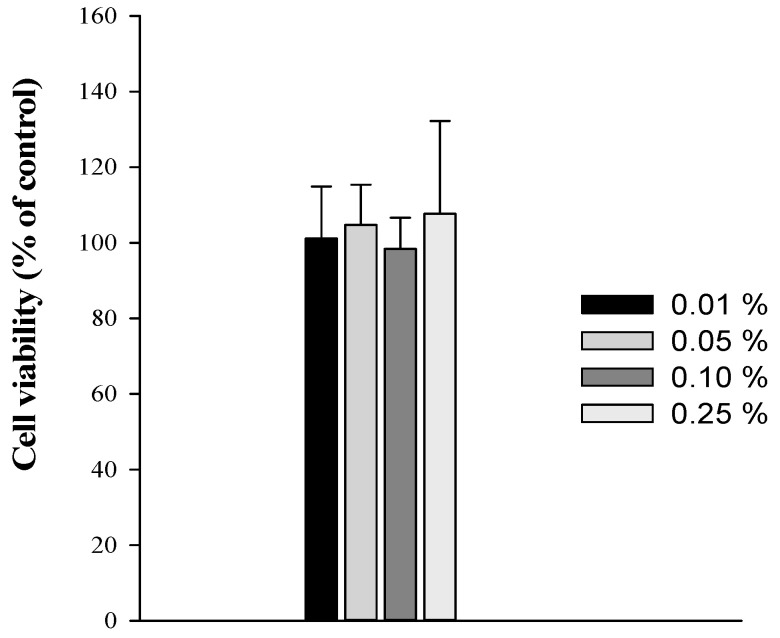
Cytotoxicity of SNEDDS without drug in Caco-2 cells (mean ± SD, *n* = 4).

The effect of SNEDDS components on the cell viability was evaluated using MTT assay in Caco-2 cells. Since SNEDDS is orally administered and Caco-2 cells are primarily used as an in vitro model for intestinal epithelium, Caco-2 cells were selected for the MTT study of SNEDDS components. As shown in Figure 3, SNEDDS without drugs (empty vehicle) did not show any cytotoxicity at the concentration range used in the preparation of SNEDDS. Furthermore, since the dilution with the GI fluids after oral administration of SNEDDS significantly attenuates the concentration of formulation components in the GI tract, the potential toxicity of SNEDDS components should be minimal.

### 3.3. Preparation and Characterization of Drug-Loaded SNEDDS

#### 3.3.1. Construction of a Pseudoternary Phase Diagram

**Figure 4:**
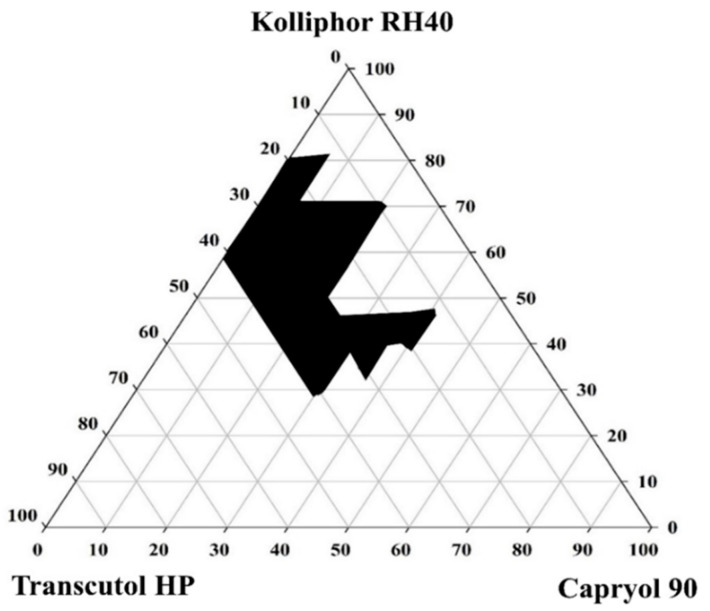
Pseudoternary phase diagram using Capryol 90 as an oil, Kolliphor RH40 as a surfactant, and Transcutol HP as a cosurfactant. The black area is the self-nanoemulsifying region.

Since surfactants and cosurfactants reduce the interfacial energy and provide a mechanical barrier against the coalescence of emulsion droplets [29], the mixing ratio of oil to surfactant and cosurfactant plays an important role in the formation of SNEDDS. To identify the self-nanoemulsifying regions and optimize the concentration of phase components in the SNEDDS, a pseudoternary phase diagram containing oil (Capryol 90), surfactant (Kolliphor RH40), and cosurfactant (Transcutol HP) was constructed as shown in Figure 4. This pseudoternary phase diagram provided a self-nanoemulsifying region where transparent emulsions were produced upon dilution. As shown in Figure 4, the black area of the constructed ternary phase indicates a nanoemulsion zone. Spontaneous emulsion formation was observed when the amount of surfactant was 30–80%, and the emulsification efficiency was good when the total concentration of the surfactant and cosurfactant accounted for more than 60% of the SNEDDS. Furthermore, the addition of the drug (2% *w*/*w*) did not significantly alter the self-emulsifying performance of the corresponding formulation.

For the in vitro characterization of SNEDDS, a series of drug-loaded SNEDDS were prepared, and their in vitro properties were evaluated as follows.

#### 3.3.2. Thermodynamic Stability

A thermodynamic stability study was performed to exclude metastable formulations. As summarized in Table 2, all of the tested formulations did not show any precipitation, cloudiness, or phase separation after centrifugation, heat–cool cycles, and freeze–thaw cycles, confirming the stability of reconstituted nanoemulsions. Therefore, the stability of SNEDDS under these stress conditions appeared to be acceptable.

#### 3.3.3. Droplet Size and Zeta Potential

The size of emulsion droplets has a great impact on the rate and extent of drug release [30]. In addition, the larger interfacial surface area of smaller particles may facilitate the diffusion of fine emulsion droplets across the unstirred water layer as well as lymphatic drug absorption from the GI tract, thereby affecting the bioavailability of drugs [30]. Therefore, the size distribution of emulsion droplets was examined for each formulation. As summarized in Table 3, all the tested formulations exhibited nano-sized droplets with average sizes less than 50 nm. In addition, an increase in cosurfactant concentrations decreased the droplet size and PDI. This may be, at least in part, because the cosurfactant could lower the interfacial tension and fluidize the hydrocarbon region of the interfacial film, decreasing the bending stress of the interface [31]. The nano-sized droplets from the developed SNEDDS could provide a large interfacial surface area for rapid drug release [13].

The zeta potential of each formulation was also evaluated since the surface charge plays an important role in colloidal stability. As shown in Table 3, all the tested formulations displayed negative charges of −2.77 ± 1.21 to −4.36 ± 0.50, probably due to free fatty acids of the oil, surfactant, and cosurfactant [32]. The electrostatic repulsion among negatively charged particles could help minimize coalescence and maintain the stable dispersion of emulsion droplets. In addition to electrostatic stabilization, non-ionic surfactants could stabilize the system sterically by forming a coat on the surface of droplets [33].

#### 3.3.4. Emulsification Time and Robustness to Dilution

The self-emulsification time is also a critical factor for the preparation of SNEDDS since the emulsification process is the rate-determining step for drug absorption. As shown in Table 3, all tested SNEDDS dispersed quickly upon aqueous dilution, and their emulsification times were short within the range of 15–75 s. The higher surfactant concentrations in the continuous phase resulted in longer emulsification times, maybe due to the increased viscosity [34]. In addition, as the concentration of cosurfactants increased, the spontaneity of the emulsification process increased, and thus, the self-emulsification time decreased. This may be due to excess diffusion of the aqueous phase into the oil as the cosurfactants reduced the interfacial tension, resulting in significant interface disruption and discharge of droplets into the aqueous phase [35]. 

After dilution with distilled water, 0.1N HCl, or phosphate buffer (pH 6.8), the resultant nanoemulsions maintained optical clarity and did not show any phase separation, cloudiness, or precipitation (Table 4).

#### 3.3.5. Selection of the Optimal SNEDDS

Among the tested formulations, SNEDDS-F4, which was composed of 20% Capryol 90, 45% Kolliphor RH40, and 35% Transcutol HP, exhibited good in vitro properties as a self-emulsifying formulation. It formed nanoemulsion quickly with a short emulsification time (~18 s) and showed optical clarity (Table 3). SNEDDS-F4 also exhibited a narrow size distribution with a mean droplet size of 17.8 ± 0.36 nm and high encapsulation efficiency (>90%). Due to the presence of free fatty acids, SNEDDS-F4 had a negative charge, which may support dispersion stability via electrostatic repulsion. Furthermore, when diluted (10, 100, and 1000 folds) with distilled water, 0.1N HCl, or phosphate buffer (pH 6.8), SNEDDS-F4 maintained a transparent nanoemulsion without any phase separation or drug precipitation over 24 h, implying its robustness to dilution in the GI tract. SNEDDS-F4 also dramatically increased the solubility of AC1497 (16.6 mg/mL). Therefore, SNEDDS-F4 was selected as an optimal formulation for further characterization.

### 3.4. Morphology

**Figure 5:**
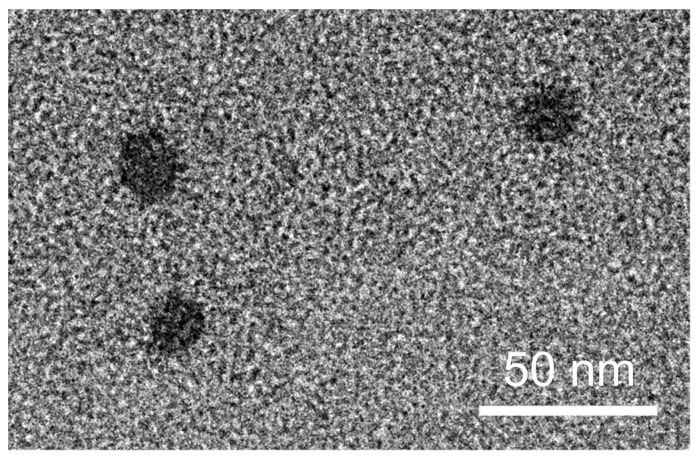
TEM image of SNEDDS-F4.

The morphological characteristics of SNEDDS-F4 were examined by TEM, as shown in Figure 5. It exhibited spherical droplets without aggregation, and its size was comparable to the size determined by dynamic light scattering.

### 3.5. In Vitro Drug Release Studies

**Figure 6:**
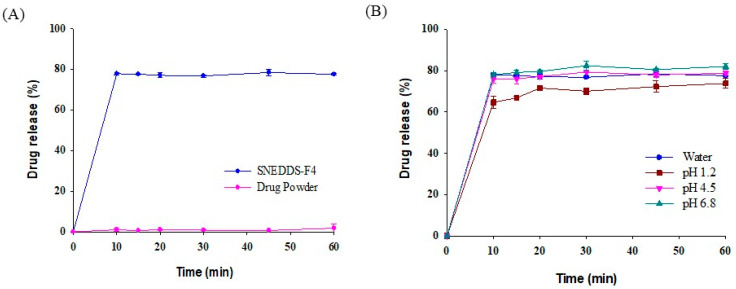
Drug release profiles of SNEDDS-F4 (mean ± SD, *n* = 3). (**A**) Comparison of SNEDDS-F4 and drug powder in water, (**B**) drug release profiles of SNEDDS-F4 at different pH conditions.

The drug release profile of SNEDDS-F4 was evaluated in comparison with that of the drug powder. As shown in Figure 6A, SNEDDS-F4 significantly enhanced the rate and extent of drug release in water, achieving approximately 80% drug release within 10 min, while drug dissolution from the drug powder was minimal (<2%). This could be because SNEDDS-F4 rapidly formed nanoemulsions upon exposure to the aqueous medium, and the large interface of the resultant nano-sized emulsion droplets facilitated the rapid drug release. Furthermore, the drug release profile of SNEDDS-F4 was not dependent on the pH (Figure 6B). SNEDDS-F4 exhibited a similarly high and rapid drug release at all pH conditions ranging from acidic to neutral, implying effective drug release along the GI tract (Figure 6B).

**Figure 7:**
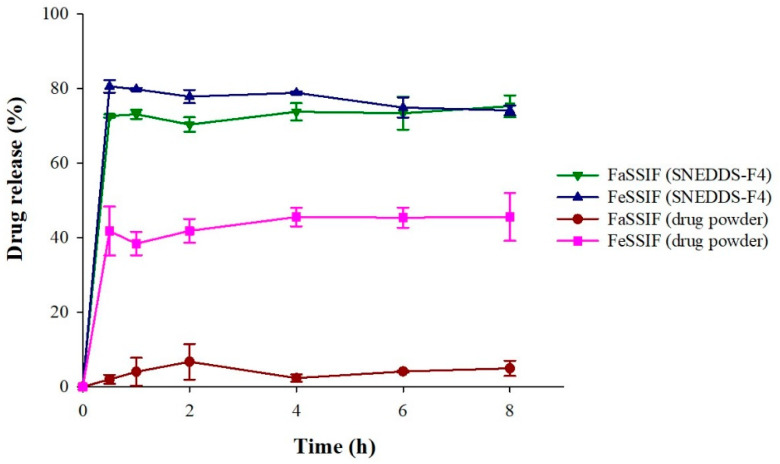
Drug release profiles of SNEDDS-F4 and drug powder in FaSSIF and FeSSIF (mean ± SD, *n* = 3).

Since the drug release from SNEDDS formulations can be affected by food intake, the drug release profiles of SNEDDS-F4 were also examined under the fasted and the fed conditions by using simulated intestinal fluids (FaSSIF and FeSSIF) [26]. As summarized in Figure 7, the dissolution of AC1497 significantly increased in the fed condition compared to that in the fasted condition. To reflect the biliary response to meal intake, FeSSIF contained considerably higher concentrations of bile salts and phospholipids than FaSSIF, which could facilitate the wetting of solids and the solubilization of lipophilic drugs into mixed micelles [26]. In addition, FeSSIF included lipid digestion products such as glyceryl monooleate and sodium oleate that could enhance the solubility of lipophilic drugs. As a result, the drug powder achieved greater drug dissolution in FeSSIF than in FaSSIF (Figure 7). In contrast, SNEDDS-F4 exhibited much higher drug release both in the fed and the fasted conditions (Figure 7) than the drug powder. SNEDDS-F4 achieved rapid drug release of 70% and 80% within 30 min in FaSSIF and FeSSIF, respectively, and maintained similarly high drug release profiles up to 8 h. These results suggest that SNEDDS-F4 is effective in improving the rate and extent of drug release and minimizing the effect of food on drug absorption.

### 3.6. Pharmacokinetic Studies

**Figure 8:**
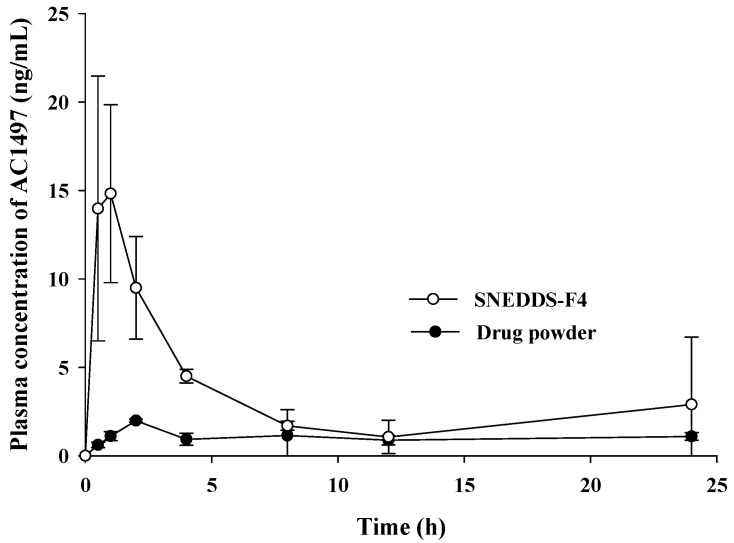
Plasma concentration–time profile of AC1497 following the oral administration of AC1497 in different formulations to rats (mean ± SD, *n* = 3). The dose was equivalent to 20 mg/kg of AC1497.

The pharmacokinetic characteristics of SNEDDS-F4 were examined after oral administration in rats. The pharmacokinetic parameters and the plasma concentration–time profiles of AC1497 are summarized in Table 5 and Figure 8. SNEDDS-F4 exhibited significantly higher oral absorption of AC1497 than the drug powder in rats. As shown in Table 5, the C_max_ and AUC of AC1497 were 6.82- and 3.14-fold, respectively, higher for the SNEDDS-F4 than for the drug powder. In addition, SNEDDS-F4 exhibited much faster drug absorption with a shorter T_max_. These results may be explained by enhanced solubility and drug dissolution via SNEDDS-F4. After oral administration, SNEDDS-F4 could rapidly form nanoemulsions upon exposure to the GI fluids. Given that lipophilic drugs display dissolution rate-limited absorption [36], the formation of nanoemulsions in the GI tract retained the drug in the solution state and bypassed drug dissolution, leading to the enhanced drug absorption. Furthermore, the nano-sized emulsion droplets could provide a large interfacial surface area for rapid drug release, facilitating drug absorption. This was also supported by the shorter T_max_ of SNEDDS-F4 than that of the drug powder. In addition, SNEDDS-F4 dramatically increased drug solubility, resulting in the enhanced drug absorption [30]. Surfactants used in the formulation may also contribute to the enhanced drug absorption by increasing mucosal permeability of intestinal epithelium [37]. On the other hand, SNEDDS-F4 exhibited a high variability of AUC (Table 5). Considering that SNEDDS formulation comes in contact with aqueous GI fluids and produces nano-sized emulsion droplets under mild agitation by GI motility, the inter-subject variability in physiological properties, including the composition and volumes of GI fluids, the intensity of GI motility, and transit time, may affect the efficiency of nanoemulsion formation, contributing to the variability in systemic drug exposure. Taken together, SNEDDS-F4 was effective in improving the oral absorption of AC1497 in rats.

## 4. Conclusions

In the present study, various SNEDDS of AC1497 were prepared by using different weight ratios of Capryol 90, Kolliphor RH40, and Transcutol HP. Among the tested formulations, SNEDDS-F4 containing 20% Capryol 90, 45% Kolliphor RH40, and 35% Transcutol HP exhibited good self-emulsification efficiency and produced nano-sized emulsion droplets with high drug encapsulation efficiency. SNEDDS-F4 achieved much faster and higher drug release than the drug powder. In addition, SNEDDS-F4 exhibited a similarly high and rapid drug release at all pH conditions ranging from acidic to neutral, implying effective drug release along the GI tract. Accordingly, SNEDDS-F4 exhibited significantly enhanced oral exposure of AC1497 in rats. These results suggest that SNEDDS-F4 should be effective in improving the oral bioavailability of AC1497, a poorly water-soluble drug.

## Figures and Tables

**Table 1:** Emulsification efficiency of various surfactants and cosurfactants (mean ± SD, *n* = 3).

	No. of Inversions	% Transmittance
**Surfactants**		
Solutol HS 15	24	90.25 ± 1.27
Tween 80	10	71.60 ± 6.41
Labrasol	17	13.48 ± 3.09
Kolliphor ELP	28	97.92 ± 0.48
Kolliphor RH40	28	99.62 ± 0.40
Brij L4	8	22.79 ± 6.92
**Cosurfactants**		
Campul PG8	47	97.78 ± 1.32
PEG 400	22	100.3 ± 0.50
Ethanol	17	100.6 ± 0.22
Propylene Glycol	18	100.7 ± 0.16
Transcutol HP	12	100.4 ± 0.12

**Table 2:** Thermodynamic stability of the drug-loaded SNEDDS.

SNEDDS	Ratio *	Centrifugation	Heat–Cool Cycles	Freeze–Thaw Cycles
F1	20:70:10	Stable	Stable	Stable
F2	20:60:20	Stable	Stable	Stable
F3	20:50:30	Stable	Stable	Stable
F4	20:45:35	Stable	Stable	Stable
F5	25:45:30	Stable	Stable	Stable
F6	25:40:35	Stable	Stable	Stable
F7	30:40:30	Stable	Stable	Stable
F8	30:35:35	Stable	Stable	Stable

*: Oil/Surfactant/Cosurfactant (*w*/*w*/*w*).

**Table 3:** Particle size, PDI, zeta potential, encapsulation efficiency (EE), self-emulsification time, and transmittance (%) of the drug-loaded SNEDDS (mean ± SD, *n* = 3).

SNEDDS	Size (nm)	PDI	Zeta Potential (mV)	EE (%)	Emulsification Time (sec)	Transmittance (%)
F1	35.0 ± 2.86	0.87 ± 0.06	−4.18 ± 0.35	85.3 ± 6.03	75	72.2 ± 5.06
F2	24.0 ± 5.90	0.51 ± 0.26	−3.84 ± 0.92	91.5 ± 1.65	44	74.4 ± 0.59
F3	21.5 ± 1.54	0.35 ± 0.03	−4.10 ± 1.29	90.4 ± 0.58	40	85.5 ± 2.75
F4	17.8 ± 0.36	0.14 ± 0.04	−4.36 ± 0.50	93.6 ± 2.28	18	88.6 ± 0.89
F5	22.3 ± 0.57	0.33 ± 0.01	−3.98 ± 0.67	92.0 ± 2.14	25	87.0 ± 1.17
F6	18.6 ± 0.76	0.03 ± 0.01	−3.05 ± 0.18	89.8 ± 1.01	15	84.3 ± 2.26
F7	20.8 ± 0.66	0.04 ± 0.02	−2.80 ± 0.11	92.6 ± 0.44	17	87.3 ± 1.69
F8	22.3 ± 0.79	0.03 ± 0.01	−2.77 ± 1.21	90.1 ± 0.43	16	82.7 ± 0.49

**Table 4:** Robustness to dilution of the drug-loaded SNEDDS.

SNEDDS	Distilled Water			0.1 N HCl			Phosphate Buffer (pH 6.8)		
	10	100	1000	10	100	1000	10	100	1000
F1	Clear	Clear	Clear	Clear	Clear	Clear	Clear	Clear	Clear
F2	Clear	Clear	Clear	Clear	Clear	Clear	Clear	Clear	Clear
F3	Clear	Clear	Clear	Clear	Clear	Clear	Clear	Clear	Clear
F4	Clear	Clear	Clear	Clear	Clear	Clear	Clear	Clear	Clear
F5	Clear	Clear	Clear	Clear	Clear	Clear	Clear	Clear	Clear
F6	Clear	Clear	Clear	Clear	Clear	Clear	Clear	Clear	Clear
F7	Clear	Clear	Clear	Clear	Clear	Clear	Clear	Clear	Clear
F8	Clear	Clear	Clear	Clear	Clear	Clear	Clear	Clear	Clear

Where “clear” means no change in clarity without phase separation or precipitation.

**Table 5:** Pharmacokinetic parameters of AC1497 following the oral administration of AC1497 in different formulations to rats (mean ± SD, *n* = 3). The dose was equivalent to 20 mg/kg of AC1497.

Parameters	Pure Drug	SNEDDS-F4
C_max_ (ng/mL)	2.30 ± 0.44	15.7 ± 6.40 *
T_max_ (h)	4.0 ± 3.5	0.8 ± 0.3 *
AUC (ng*h/mL)	25.0 ± 6.26	78.5 ± 38.9 *

*: *p* < 0.05, compared to the control group (pure drug).

## Data Availability

Not applicable.
